# A novel transition clinic structure for adolescent and young adult patients with childhood onset rheumatic disease improves transition outcomes

**DOI:** 10.1186/s12969-021-00651-w

**Published:** 2021-12-01

**Authors:** Rebecca S. Overbury, Kelly Huynh, John Bohnsack, Tracy Frech, Aimee Hersh

**Affiliations:** 1 Division of Pediatric Rheumatology, University of Utah, 30N 1900E 4B200, Salt Lake City, UT 84132 USA; 2 Division of Rheumatology, University of Utah, 30N 1900E 4B200, Salt Lake City, UT 84132 USA; 3 Intermountain Healthcare, Salt Lake City, UT USA

**Keywords:** Transition of care, Healthcare transition, Pediatric rheumatology, Juvenile idiopathic arthritis, Systemic lupus erythematosus, Adolescent medicine, Healthcare process, Transition readiness, Transition readiness assessment, Loss to follow-up

## Abstract

**Background:**

The transition of health care from Pediatric to Adult providers for adolescents and young adults with chronic disease is associated with poor outcomes. Despite the importance of this transition, over 80% of these patients do not receive the services necessary to transition to Adult health care. In 2018, we initiated a transition clinic structure, integrating an Internal Medicine - Pediatrics trained Adult Rheumatologist in a Pediatric Rheumatology clinic to guide this transition. Our goal was to improve transition outcomes. We report the methods of this clinic and its preliminary outcomes.

**Methods:**

For patients referred to the transition clinic, the Adult Rheumatologist assumed medical management and implemented a six-part modular transition curriculum. This curriculum included a Transition Policy, Transition Readiness Assessment, medication review and education, diagnosis review and education, and counseling on differences between Pediatric and Adult-oriented care. Eligible patients and their families were enrolled in a prospective observational outcomes research registry. Initial data from this transition clinic is reported including adherence with certain aspects of the transition curriculum and clinic utilization.

**Results:**

The transition clinic Adult Rheumatologist saw 177 patients in 2 years, and 57 patients were eligible for, approached, and successfully enrolled in the registry. From this registry, all patients reviewed the Transition Policy with the Adult Rheumatologist and 45 (78.9%) completed at least one Transition Readiness Assessment. Of the 22 patients for whom transition was indicated, all were successfully transitioned to an Adult Rheumatologist. 17 (77.3%) continued care post-transition with the transition clinic Adult Rheumatologist, and 5 (22.7%) continued care post-transition with a different Adult Rheumatologist. The median time between the last transition clinic visit and first Adult clinic visit was 5.1 months.

**Conclusions:**

Our experience demonstrated the success of our clinic model regarding participation in the transition curriculum and improved clinic utilization data. Our results are an improvement over transition rates reported elsewhere that did not implement our model. We believe that this structure could be applied to other primary care and subspecialty clinics.

**Trial registration:**

This research was approved by the University of Utah Institutional Review Board (IRB) in January 2019 (IRB_00115964). Patients were retrospectively registered if involved prior to this date.

**Supplementary Information:**

The online version contains supplementary material available at 10.1186/s12969-021-00651-w.

## Background

Every year, thousands of adolescent and young adult (AYA) patients are expected to transfer their medical care from a Pediatric doctor to an Adult doctor. These patients include AYA previously diagnosed with chronic medical conditions in childhood, such as childhood onset rheumatic disease (CORD). The process of preparing for this transfer of care is known as health care transition (HCT). This need to transfer providers can lead to a disruption of medical care for reasons inherent to the health care system as well as challenges faced by AYA. Evidence strongly supports the need for a structured HCT process for AYA patients to improve the likelihood of continuous care [1, 2].

Adolescence or young adulthood is ideally the stage of development when dependence on the family unit evolves into independence. This evolution includes emotional, cognitive, physical, and sexual development and is not complete until halfway through the third decade of life or beyond [3, 4]. Ultimately, these changes should provide the skill set needed to navigate as a successful adult.

This evolution and development can be complicated by the patient’s chronic disease [5]. AYA patients are more likely to experience threats to normal development compared to their healthy peers, and they often exhibit behaviors which risk control of a previously well-managed chronic disease. In addition to the routine struggles of adolescence and young adulthood, these AYA may have an altered physical appearance from disease manifestations or medication side effects, physical limitations, or hospitalizations and frequent medical visits which can impede the successful completion of education and other life goals [5, 6].

The path to independence for AYA patients with a chronic disease diagnosis also includes challenges within the health care system. In the United States, the subspecialty health care system is largely split between Child-oriented care systems and Adult-oriented care systems. As a result, AYA patients must often navigate a new health care system and may experience changes in their health insurance status. There are also cultural differences between Adult-oriented and Pediatric care, including expectations of self-management, diminished role for family members, fewer on-site ancillary services, and stricter clinic rules and policies. These differences may be intimidating to the AYA patient seeking to receive their care in a new Adult clinic model. These expectations also often exceed the skills of the AYA patient’s limited experience in self-management and increase the risk for gaps in continuity of care and poor disease outcomes [7].

In addition to personal risk to the patient, gaps of care negatively affect the productivity of health care facilities and patients’ timely access to medical services. This often manifests as under-utilization of planned health care services, including no-show visits [8, 9]. A recent systematic review in 2020 found that no-show rates in specialty clinics average 19% [8]. Young age has been found to be a positive predictor for these no-show visits [10, 11], putting AYA in a group at high risk for this behavior. These gaps in care during HCT can result in a loss of access to a doctor and prescription medications, and consequent high rates of unplanned health care utilization such as urgent care visits, emergency room visits, or hospitalizations for disease flares [12]. Additionally, the successful AYA clinic attendance with an Adult provider post-transition after leaving the Pediatric health care system, which is a well-recognized metric of a successful HCT [13], has been found to be as low as 60% at 6 months and 33% at 3 months [14, 15].

The American College of Physicians (ACP), the American Academy of Pediatrics (AAP), and the American Academy of Family Physicians (AAFP) recognize the importance of HCT for AYA, and have produced consensus guidelines for HCT [2]. As a result, some academic centers have established programs for facilitating HCT. However, the best HCT models to accomplish these recommendations have not yet been determined. Additionally, the vast majority of AYA are not currently receiving the interventions outlined in these guidelines. For example, data from the National Survey of Children’s Health (2018–2019) in the state of Utah (where this research was conducted) found that only 10.7% of all patients aged 12–17 received the services necessary for transition to Adult health care, and only 11.5% for those with special health care needs, such as CORD. Similar data is only marginally better nationwide with 16.9% of all patients aged 12–17 receiving these necessary services and only 22.9% of those with special health care needs.

Recognizing this gap in care, in November 2018 the Divisions of Pediatric Rheumatology and Rheumatology, in the University of Utah Departments of Pediatrics and Internal Medicine respectively, collaborated to initiate a transition clinic structure named ACCORD (the Adult Center for Childhood Onset Rheumatic Disease).

This clinic structure and intervention is based on successful models elsewhere, and systematic reviews, both in Rheumatology and other sub-specialty clinics [16–18]. Data is scant regarding how to evaluate HCT models in practice, and holistic evaluations of the HCT process are limited [18]. However, common identified metrics of a successful HCT include attendance with a first Adult provider and the gap between the last Pediatric visit and the first Adult visit [13]. Identified design features that are important for successful HCT include personalized transition timing, having a transition education component, joint clinic structures with Pediatric and Adult providers, and the presence of a transition facilitator [19]. Finally, while randomized controlled trials are the standard for testing any intervention, based on expert opinion, it is no longer ethical to deny AYA access to HCT in this pursuit [18]. Based on this evidence, the structure of ACCORD was built to integrate an Internal Medicine - Pediatrics trained Adult Rheumatologist in a Pediatric Rheumatology clinic to initiate and guide a transition education curriculum and to serve as a transition facilitator for the HCT of AYA patients with CORD.

While not all of our aspirational outcomes are measured or presented here, ACCORD ultimately seeks to incorporate priorities in HCT which have been identified in qualitative studies elsewhere including the chance to develop trust with their new doctor, and achievement of balanced family involvement that is supportive but not smothering [16, 17, 20–22]. The overarching goal of the ACCORD clinic is to improve individual HCT experience, improve populational health outcomes, and reduce per capita cost of care. We also hope to define the metrics of a successful HCT and improve long term outcomes in patients with CORD. The purpose of this paper is to report the methods and process of the ACCORD clinic as well as our experience with our initial cohort of patients.

## Methods

### Description of the ACCORD clinic

The University of Utah is in Salt Lake City, Utah in the United States. The University has a Division of Rheumatology in the Department of Internal Medicine and a Division of Pediatric Rheumatology in the Department of Pediatrics. These Divisions operate separate research, clinical, and ancillary services. The Division of Pediatric Rheumatology provides care in Primary Children’s Hospital where both professional and facility services are contracted with almost all area health plans. The Division of Rheumatology operates its clinics at the University of Utah Hospital and Clinics, where professional and facility services are contracted with a smaller subset of area health plans. This discrepancy in area health plan coverage between Pediatric Rheumatology and Rheumatology presents a barrier to HCT for AYA in our system, as it can necessitate transfer of care to an adult provider outside of the University of Utah Health Care system.

The ACCORD clinic is held once a week in the Pediatric Rheumatology clinic at Primary Children’s Hospital and is staffed by an Adult Rheumatologist (R.O.), a Board Certified Rheumatologist who is Board Certified in both Internal Medicine and Pediatrics. Patients are eligible for referral to the ACCORD clinic once they are 16 years of age or older. Patients are referred to the ACCORD clinic either by an external referral, for a patient with a possible Rheumatic disease, or by an internal referral from a Pediatric Rheumatologist who has been managing an AYA patient with CORD. Therefore, some patients scheduled in the ACCORD clinic have a diagnosis of a CORD (for example, juvenile idiopathic arthritis [JIA] or juvenile onset systemic lupus erythematosus [SLE]), some patients have adult-onset rheumatic disease (diagnosed at the age of 16 years or older), and some do not have a rheumatic disease diagnosis. An advantage of holding the ACCORD clinic in the Pediatric Rheumatology space is that it allows Pediatric Rheumatologists to introduce the ACCORD clinic Adult Rheumatologist to patients and families up to several years prior to referral to the ACCORD Clinic. Another advantage is that practically all third-party payers are willing to reimburse for HCT care at this site, including visits with the Adult Rheumatologist in the ACCORD clinic. Patients transition their medical care entirely to the Adult Rheumatologist in the ACCORD clinic once the handoff has been made. Patients and their families are informed that in addition to transition planning, disease activity assessment and medication management will be addressed at every ACCORD clinic visit.

### Transition preparation in the ACCORD clinic

The main purpose of the ACCORD Clinic is to prepare AYA patients to assume full responsibility for their disease management. Parents can accompany the patient to the clinic visit, but at least some, and eventually all, of the clinic visit is performed without the parent present. HCT planning curriculum is reviewed at each ACCORD clinic visit. Patients can schedule as many consecutive visits as necessary in the ACCORD clinic. Specific plans are made for the patient to transition to an Adult Rheumatology clinic once the patient, family, and Adult Rheumatologist are prepared and comfortable. Some patients transfer to the Adult Rheumatologist’s (R.O.) clinic in the University of Utah’s Adult Rheumatology clinic. But arrangements to transition to other Adult Rheumatologists are also made for a variety of reasons, including personal choice, a provider closer to the patient’s home, or because the University of Utah Adult Rheumatology providers are not on the patient’s health plan panel.

During their visits in the ACCORD clinic, patients and their families undergo education and guided conversations to prepare for HCT and to gauge transition readiness of the patient and the family. The basis of this education is the evidence-based work of Got Transition®, last updated in 2020, specifically the Six Core Elements for Transitioning Youth to an Adult Health Care Clinician [23] . Transition planning and education encompasses some or most of the clinical visit, depending on need, and is broken down into six discrete modules (Table 1). The entirety of this transition education is conducted by the Adult Rheumatologist. These modules are reviewed in person or distributed via personal email, to be reviewed at subsequent encounters. They are completed longitudinally, generally progressing to the next module with each consecutive visit. No metrics are collected regarding outcomes related to individual modules, such as disease activity, or pre or post surveys or testing.

**Table 1:** HCT curriculum and transition modules

Module	Target age	Patient materials and data	Parent materials and data
1	(12–14 yo)	o Introductory sheet o Transition policy	o Introductory sheet o Transition policy
2	(14–18 yo)	o Plan of care o Frequently asked questions … o Transition readiness assessment	o Transition readiness assessment
3	(14–18 yo)	o Medical summary and emergency care plan o Medication list	
4	(18–21 yo)	o “10 things to know” o HAQ o Mind the Gap o PHQ 9 o SF 36	o Mind the Gap
5	(18–26 yo)	o Medical summary and emergency care plan o Medication list o Plan of care o Transition readiness assessment o Transfer Letter template	o Transition readiness assessment
6	≤ 12 months from 1st visit with Adult Rheumatology	o Transition feedback survey o PROMIS® Self-Efficacy for Managing Chronic Conditions-Medications and Treatments	o Transition feedback survey

*HCT* health care transition, *yo* years old, *HAQ* Stanford Health Assessment Questionnaire, *PHQ 9* Patient Health Questionnaire 9, *SF 36* RAND Short Form 36, *PROMIS* Patient-Reported Outcomes Measurement Information System

#### Module 1

The first module focuses on a review of the Pediatric Rheumatology Transition Policy which was formulated and approved by R.O. and all providers in the Division of Pediatric Rheumatology. This module focuses on the goals of the transition process, and the age at which a patient will be expected to transfer. This module explicitly outlines the expectation that patients spend a portion of each clinical visit alone with their provider and provides direction for release of information at the age of 18 if the patient wants family members’ continued involvement in their care (Additional File 1).

#### Module 2

The second module focuses on a Plan of Care (Got Transition®), Frequently Asked Questions (Got Transition®), and Transition Readiness Assessment (Got Transition®). The Plan of Care document is a paper document that provides AYA the opportunity to reflect upon and plan for anticipated barriers and goals of transition which can guide both current and future conversations. This is completed by the AYA, with an open invitation to share this exercise with family members or the Adult Rheumatologist if the patient desires. The Frequently Asked Questions document covers many of the anticipated questions that AYA or their families may have as they prepare for transition; again, to guide conversation. The Transition Readiness Assessment is a survey to gauge perspectives regarding the patient’s current skill set for autonomy in the Adult health care system. There is a patient-oriented survey, completed by the patient, and a parent-oriented survey, completed by the parent or guardian. The Transition Readiness Assessment surveys are completed twice, once shortly after entry into the ACCORD clinic, and then a second time (Module 5) just prior to the transition to an Adult Rheumatologist.

#### Module 3

The third module includes a Portable Medical Summary (Got Transition®) and a medication list. The Portable Medical Summary, which is co-developed by the patient and the Adult Rheumatologist, is a paper document providing the patient a customizable template to document their medical history and objective medical information. This exercise serves as an educational tool to familiarize the patient with aspects of their medical history that they may not have previously been expected to recall or understand. It also provides, once complete, a portable and concise paper document summarizing their past medical history and current medical needs; an especially valuable tool for AYA patients who frequently travel, are away at college, or are without a stable primary care provider. The Portable Medical Summary is also a valuable tool and medical summary for adult providers who will be assuming the care of the patient in the future. The medication list is an exercise to gauge the patient’s ability to recall their medications from memory. This is also an opportunity to review the logistics of medication refills, the indication for medications, and how to administer one’s own injected medications if this responsibility has not yet been transitioned to the AYA patient.

#### Module 4

The fourth module includes 10 Things to Know, an institution specific document formulated and approved by R. O and all providers in the Division of Pediatric Rheumatology that outlines some of the important ways in which a relationship with an Adult Rheumatology provider may differ from that with a Pediatric Rheumatology provider. The text of this document was formulated to reflect an 8th grade reading level. An additional file shows this document in more detail (Additional File 2). This module also focuses on collecting data regarding the patient’s current state of function and well-being, utilizing the Stanford Health Assessment Questionnaire (HAQ), the Patient Health Questionnaire-9 (PHQ-9), and the RAND Short Form-36 (SF-36). These patient-reported outcomes are explained to the patient, so that they have a clear understanding of how these are used to assess disease activity.

#### Module 5

The fifth module is intentionally redundant and includes a review and possible revision of the Portable Medical Summary, the medication list, a review of contraception planning, the Plan of Care document, and repeat assessments using the Transition Readiness Assessment surveys. The purpose of this module is to update these metrics in the context of an anticipated and imminent transition to Adult-oriented care. Both the Transition Readiness Assessment and Portable Medical Summary are included in the eventual documents transferred to the accepting Adult Rheumatologist.

#### Module 6

The sixth and final module occurs after transition to the Adult Rheumatology clinic. This module is sent via personal email and includes the HCT Feedback Survey (Got Transition®) that elicits feedback on the HCT process, both from the patient and from the family. This module also includes the PROMIS Item Bank v1.0 - Self-Efficacy for Managing Chronic Conditions - Managing Medications and Treatment - Short Form 8a to quantity the patient’s confidence in independently managing their chronic condition post-HCT.

These modules are completed sequentially from the time of entry into the ACCORD clinic until the transition to an Adult Rheumatologist. Tools for each module can be completed in person or via personal email, to then be reviewed at subsequent clinic encounters. Currently, none of these modules or tools are collected or recorded in our electronic medical record. Using REDCap, electronically formatted versions of these surveys are manually entered or distributed to the patients’ and their families’ personal email addresses and results are securely stored.

Patient-specific progress towards completion of these modules is documented in the patient’s clinic note alongside other aspects of the assessment and plan. This documentation allows for communication internal to the Division of Pediatric Rheumatology, ensures longitudinal follow up and timely completion of all modules, and becomes a component of their medical record that will be forwarded to the Adult Rheumatologist. Additionally, transition planning is included as an ICD-10 code (Z71.89) for these clinic encounters, both for future research purposes and cost analysis; which is not analyzed or presented in this current manuscript.

### Prospective observational study of Transition outcomes

As mentioned above, some patients in the ACCORD clinic are eligible for the ACCORD prospective observational study of transition outcomes registry which is designed to accomplish two goals: 1) research the effect of this clinic structure on HCT outcomes, and 2) research the short and long-term outcomes of patients with CORD. Therefore, only patients who have CORD or who are diagnosed with a rheumatic disease in the ACCORD clinic are given the opportunity to enroll. Eligibility criteria otherwise include being between the ages of 12–26 years of age. As of April 2021, no eligible patients have declined to enroll. This registry collects patient identifiers, demographic data, disease specific data, the Transition Readiness Assessment surveys, the HAQ, the PHQ-9, the SF-36, the HCT Feedback Surveys, the PROMIS Item Bank v1.0 - Self-Efficacy for Managing Chronic Conditions - Managing Medications and Treatment- Short Form 8a, the date of the last visit in the ACCORD clinic, and the date of the first visit in an Adult Rheumatology clinic. This registry does not track completion of the other metrics outlined above. This data is stored in a REDCap database managed by the University of Utah.

This research is in compliance with the Helsinki Declaration and is approved by the IRB at the University of Utah (IRB_00115964) including the provision of written informed consent, parental permission, and assent as indicated.

### Statistical comparisons

Demographic data was calculated for all patients seen in the ACCORD clinic and separately for those enrolled in the registry (age, reported race, reported ethnicity, diagnosis, biologic sex). Clinical utilization data was reported and compared for the ACCORD clinic and the Pediatric Rheumatology clinic, using “N^− 1^” Chi-squared test. Preliminary data regarding transition curriculum, transition planning, and transition outcomes are reported. An alpha level of .05 was defined as significant.

## Results

### ACCORD clinic patient demographics

The ACCORD clinic began caring for patients in November of 2018, and 177 unique patients were seen in the clinic in 375 encounters by December 31, 2020. 43 (26.7%) were previously diagnosed with CORD by a Pediatric Rheumatologist (an internal referral). 73 (41.2%) had at least one subsequent return visit in the ACCORD clinic at the time of this reporting. The median age at the time of their first visit was 17 years (SD = 1.8, range 14–27). Most (78.5%) were female, and 88.7% reported their race to be White while 13.6% reported their ethnicity to be Hispanic (Table 2). The most common diagnosis was JIA, followed by SLE.

**Table 2:** ACCORD transition clinic demographics, all patients

	Patients		Visits	
	Count	%	Count	%
TOTAL	177		375	
Sex				
Female	139	78.5	307	81.9
Male	38	21.5	68	18.1
Self-Reported Race				
American Indian or Alaska Native	1	0.6	1	0.3
Asian	3	1.7	6	1.6
Black or African American	7	4.0	19	5.1
Multiple	1	0.6	1	0.3
Native Hawaiian or Pacific Islander	3	1.7	14	3.7
Patient Declined	1	0.6	4	1.1
Unavailable	4	2.3	6	1.6
White	157	88.7	324	86.4
Self-Reported Ethnicity				
Hispanic, Latino, or Spanish Origin	24	13.6	55	14.7
Not Hispanic, Latino, or Spanish Origin	150	84.8	315	84.0
Unavailable	3	1.7	5	1.3
Age in Years at Time of First Visit				
14			2	0.5
15			2	0.5
16			76	20.3
17			120	32.0
18			51	13.6
19			43	11.5
20			49	13.1
21			21	5.6
22			5	1.3
23			4	1.1
25			1	0.3
27			1	0.3

### No-show rates

There was no statistical significant difference in no-show rates between the ACCORD clinic and the general Pediatric Rheumatology clinic; both for patients of all ages, or for those limited to ≥16 years of age (Table 3).

**Table 3:** Clinic no-show rates

	ACCORD clinic % (***n***)	ACCORD clinic ≥ 16 years old % (***n***)	General Pediatric Rheumatology clinic % (***n***)	General Pediatric Rheumatology clinic ≥ 16 years old % (***n***)
Total no-show encounters	16.0 (69)	15.2 (56)	15.6 (1458)	15.4 (303)

No-show encounters = no-show encounters + same day cancelations

Denominator is # of patients expected to be seen/scheduled in clinic 24 h prior to clinic start

No-show encounters *X*^*2*^ *[1, N = 2045] = 0.043, p = .83*

≥ 16 years of age, no-show encounters *X*^*2*^ *[1, N = 1983] = 0.008, p = .93*

### Transition registry

57 of the 177 patients seen in the ACCORD clinic between November 2018 and December 31, 2020, had been approached to consent to enroll in the registry for the prospective observational outcome registry. In addition to exclusion criteria (specifically not having a rheumatic diagnosis), screened patients were not enrolled as of the time of this publication if they were only seen in ACCORD for a single visit before HCT, were lost to follow up prior to transition, or were new to the ACCORD clinic and not yet been enrolled. Patients with suspected disease, but without rheumatic diagnosis, were followed in the ACCORD clinic but not enrolled. Patients without suspected disease or rheumatic diagnosis were not followed in the ACCORD clinic and were not enrolled. Only 13 (22.8%) of those enrolled were introduced to the Adult Rheumatologist (R.O.) during a prior clinic visit with their Pediatric Rheumatologist. The median age of the patients in the Registry on December 31, 2020 was 20 years (SD = 1.9, range 17–27). The median age at diagnosis was 15 years (SD = 4.9, range 1–19). The median age at enrollment was 19 years (SD = 1.9, range 16–26). The biological sex, racial and ethnic composition of the registry is similar to the overall ACCORD patient population, 75.4% female, 78.9% White and 10.5% Hispanic (Table 4). The most common diagnoses (which were re-evaluated and verified via chart review by the Adult Rheumatologist) were JIA, followed by Rheumatoid Arthritis (Table 5).

**Table 4:** ACCORD patients enrolled in the prospective observational registry

	Patients	
	Count	%
TOTAL	57	
Sex		
Female	43	75.4
Male	14	24.6
Self-Reported Race		
American Indian or Alaska Native	1	1.8
Asian	3	5.3
Black or African American	3	5.3
Native Hawaiian or Pacific Islander	1	1.8
Not Reported	1	1.8
Unknown	2	3.5
White	45	78.9
Other	1	1.8
Self-Reported Ethnicity		
Hispanic, Latino, or Spanish Origin	6	10.5
Not Hispanic, Latino, or Spanish Origin	48	84.2
Unknown	2	3.5
Not Reported	1	1.8
Age at Diagnosis		
1	2	3.5
2	2	3.5
3	2	3.5
4	1	1.8
6	1	1.8
7	1	1.8
8	4	7.0
9	2	3.5
10	2	3.5
11	1	1.8
12	2	3.5
13	2	3.5
14	3	5.3
15	11	19.3
16	11	19.3
17	8	14.0
18	1	1.8
19	1	1.8

**Table 5:** Diagnoses in ACCORD patients enrolled in the prospective observational registry (*n* = 57)

Diagnosis	Number of patients n (%)
JIA	36 (63.2)
Polyarticular RF negative	10 (17.5)
Polyarticular RF positive	4 (7.0)
Oligoarticular	8 (14.0)
Extended	4 (7.0)
Persistent	4 (7.0)
Psoriatic	1 (1.8)
Enthesitis related	3 (5.3)
Systemic	2 (3.5)
Undifferentiated	0
Other	0
SLE	8 (14.0)
MCTD	2 (3.5)
RA	12 (21.1)
Spondylarthritis	1 (1.8)
Psoriatic arthritis	2 (3.5)
Crystal arthropathy*	1 (1.8)
Dermatomyositis/polymyositis/inflammatory myositis	1 (1.8)
Auto inflammatory**	2 (3.5)
APS	0
Systemic Sclerosis	0
Linear scleroderma	0
Sarcoidosis	0

*APS* antiphospholipid syndrome, *JIA* juvenile idiopathic arthritis, *MCTD* mixed connective tissue disease, *RA* rheumatoid arthritis, *RF* rheumatoid factor, *SLE* systemic lupus erythematosus

* Includes: gout

* Includes: adult-onset still’s disease and cryopyrin-associated periodic fever syndrome (CAPS)

### Transition preparation and completion

All of the patients who enrolled in the registry reviewed the Transition Policy with the Adult Rheumatologist. 45 (78.9%) of these patients completed at least one Transition Readiness Assessment; 12 (21.1%) had twice completed the Transition Readiness Assessment. Of those enrolled, 22 (38.6%) were appropriate for transition to an Adult Rheumatologist and referred to the Adult Rheumatologist’s (R.O.) Adult-oriented clinic or to an external Adult Rheumatologist. Of these, 17 (77.3%) continued care with the associated Adult Rheumatologist (R.O.) in the University of Utah Hospital Rheumatology Clinic. Through chart review or direct contact with the accepting Adult Rheumatology clinic, verification of at least one attended Adult Rheumatology clinic visit has been confirmed for all 22 patients who have transitioned. The median time between a last ACCORD clinic visit and a first Adult Rheumatology visit was 5.1 months (SD = 5.0, range 1.6–8.7).

## Discussion

The primary purpose of this paper is to present in detail the structure of our ACCORD Rheumatology transition clinic which incorporates HCT guidelines proposed by the American College of Physicians, the American Academy of Pediatrics, and the American Academy of Family Physicians. The earliest data from this cohort suggest that the clinic structure has a positive effect on transition outcomes including clinic utilization.

We demonstrate here that the ACCORD clinic is durable. Over 2 years, we have successfully cared for 177 unique patients, 57 of whom were enrolled in our HCT outcomes registry. Additionally, ACCORD has benefited patients regardless of which health care system they utilized for their Adult care, and regardless of whether they could continue care with the ACCORD clinic Adult Rheumatologist. This is a crucial detail because our clinic structure incorporates regular and structured HCT planning and readiness assessment for AYA which we hypothesize will demonstrate quantifiable and meaningful improvement in successful transition.

Transition clinic interventions have been shown elsewhere to increase the success of transfer of care to an adult sub-specialist clinic appointment [24, 25]. Previous studies have shown that non-adherence with adult sub-specialty clinic visits or loss to follow up following a standard transfer of care from Pediatric to Adult sub-specialist is 20–58% [26–29]. The median time between the last Pediatric Rheumatology clinic visit and the first Adult Rheumatology clinic visit is reported elsewhere to be 8–11 months [30, 31]. Our data shows improved successful transition rates during the critical stage following the last ACCORD clinic visit and the first Adult Rheumatology visit. Specifically, patients in our ACCORD Registry show a 100% successful transition rate (as defined by one scheduled and successful Adult Rheumatology clinic visit following transition) with a median of 5.1 months between the last ACCORD clinic visit and the first Adult Rheumatology clinic visit. This is an improvement over the standard of care and is important because a longer interval between the last Pediatric clinic visit and the first Adult clinic visit has been associated with medication non-adherence [30]. A weakness of these results is that we lack similar transition data for our Pediatric Rheumatology patients who choose not to attend the ACCORD transition clinic. This limits our ability to compare our results with other health care systems. Nonetheless, these preliminary results suggest improved transition rates and decreased transition intervals.

One issue demonstrated by this data is the problem of under-utilized scheduled health care, specifically no-show visits. The no-show rate for patients in this study was 15–16%, which is comparable to rates described for specialty clinics elsewhere [8]. Interestingly, this rate did not differ between younger patients and those ≥16 years of age, nor between patients of the Pediatric clinic versus the ACCORD clinic. This could reflect a replicable “baseline” or accepted “normal” no-show rate. However, as data consistently shows that no-show rates are inversely proportional to age, with young adults having the highest probability of no-show appointments, and that in Pediatric clinics the likelihood of no-show appointments increases with age, this could also be evidence of a trend towards reduced no-show rates in our ACCORD Registry [32]. Regardless, this highlights an area in need of further investigation. Arguments have been made for “treat to target” interventions which could use risk factors for non-adherence to guide interventions which could lower no-show rates [24]. Additionally, some studies have shown improvements in no-show rates with interventions such as telephone or other reminders [9]. In the future, we plan to evaluate the effect of the ACCORD clinic on no-show rates post-transition, compared to controls. Future ACCORD policy and research will be need to be directed toward this problem as this issue is particularly problematic during this crucial HCT process.

Other limitations of this research must include the possibility for bias in the ACCORD prospective observational study of transition outcomes registry. Currently, there is no systematic method for which patients are referred to the ACCORD clinic, potentially limiting the sample to those who might be more adherent at baseline or who tend to utilize appropriate health care services more regularly. Additionally, not every ACCORD patient can be captured to consider entry into the registry (if they are lost to follow up or transition to an Adult Rheumatologist before informed consent can take place.) It is not clear currently, how this may be confounding our results. We also recognize that there is a lack of diversity in our current cohort. Future directions of this research include how best to expand our HCT process to underserved and high risk populations.

Finally, our current ACCORD clinic and health care model depends on one Adult Rheumatologist. While we hope to systematize and integrate this health care model into our Pediatric Rheumatology practice more generally, this is a current weakness in the generalizability of this intervention as well as a vulnerability for the success of the ACCORD model moving forward.

While this is conjecture, our experience leads us to believe that it is the joint nature of this clinic, incorporating an Adult Rheumatologist into the Pediatric Rheumatology setting, which has contributed to the success of this clinic model. We hypothesize that the opportunity for the AYA to become familiar with and trust an Adult Rheumatologists eases the stress and hesitancy of transitioning to Adult-oriented care. Success is also likely due to the deliberate nature of the ACCORD clinic, dedicated to the HCT curriculum and planning, which appropriately sets the expectations of the patient and the patient’s family that the HCT will be completed and that it is of significant value.

In the future, we plan to offer additional results from this ACCORD transition clinic structure, and its registry, through the triple aim framework of population health, patient experience, and cost of care. Our preliminary data addresses metrics pertinent to population health, but future data analysis will also include pertinent findings relative to the patient and family experience as well as how this structure relates to or affects the cost of care of these complex patients. In the future, we intend to develop approaches for answering similar questions in our patients who do not utilize the ACCORD clinic and are not enrolled in the ACCORD registry. Future aims also include defining successful HCT and the role of a longitudinal relationship with the Adult sub-specialist in this success.

We hope to continue to advance our interventions with aims focused on physician-oriented and patient-oriented outcomes as well as to develop risk assessment tools for transitioning AYA patients. Our goal is to improve both short and long term medical and psychosocial health in AYA patients with CORD as well as the HCT process for all patients and their families.

## Conclusions

Our experience demonstrates a successful transition clinic model. We succeeded in transition rates of 100% and a median transition interval of 5.1 months. This is an improvement over transition rates reported elsewhere that did not implement our model. We believe this structure could be successfully applied to other primary care and subspecialty clinics.

## Supplementary Information

**Additional file 1.** Transition Policy

**Additional file 2.** 10 Things to Know about Adult Rheumatology Clinic

## Data Availability

The datasets used and/or analyzed during the current study are available from the corresponding author on reasonable request.

## Abbreviations

AAFP: American Academy of Family Physicians
AAP: American Academy of Pediatrics
ACCORD: Adult Center for Childhood Onset Rheumatic Disease
ACP: American College of Physicians
AYA: Adolescents and young adults
AYA-CORD: Adolescents and young adults with childhood onset rheumatic disease
CORD: Childhood onset rheumatic disease
HAQ: Stanford Health Assessment Questionnaire
HCT: Health care transition
IRB: Institutional Review Board
JIA: Juvenile Idiopathic Arthritis
PHQ 9: Patient Health Questionnaire 9
PROMIS: Patient-Reported Outcomes Measurement Information System
SLE: Systemic Lupus Erythematosus
SF 36: RAND Short Form 36
