# Supplementary material for: A novel transition clinic structure for adolescent and young adult patients with childhood onset rheumatic disease improves transition outcomes
Source: Pediatr Rheumatol Online J. 2021 Dec 1;19:164. doi: 10.1186/s12969-021-00651-w (PMC8638174; doi:10.1186/s12969-021-00651-w)
Supplement: Supplementary file 1 — Additional file 1. Transition Policy [file 12969_2021_651_MOESM1_ESM.docx]

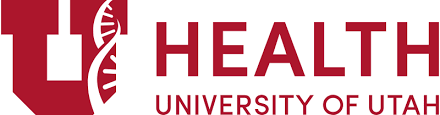


Transition Policy

We want to help our patients make a smooth transition from child-centered care to adult health care. This means we will work with teenagers, young adults and their families to help them prepare for adult health care. This process may start as early as 12 years of age. In child-centered health care, parents make most of the decisions. In adult health care, the young adult is the decision maker. To help get ready for adult care, we will spend time during some visits with the teen without the parent present. This allows teens to become more independent with their own health care.

Teens become legal adults at age 18 years. Many of our young adult patients still want their family members to help make health care decisions. We encourage this family support. In order for us to share health information with family members, the young adult needs to give us permission. Some young adults are not able to make health care decisions. In that case, we help families to legally make decisions for the young adult.

We will help young adults and their families decide on the age to change to an adult provider. The best age to switch to adult care will be different from person to person. Usually this change will occur by the age of 26 years. We will help with selecting an adult provider and sending medical records.

If you have any questions or concerns, please call us.
