# Supplementary material for: A novel transition clinic structure for adolescent and young adult patients with childhood onset rheumatic disease improves transition outcomes
Source: Pediatr Rheumatol Online J. 2021 Dec 1;19:164. doi: 10.1186/s12969-021-00651-w (PMC8638174; doi:10.1186/s12969-021-00651-w)
Supplement: Supplementary file 2 — Additional file 2. 10 Things to Know about Adult Rheumatology Clinic [file 12969_2021_651_MOESM2_ESM.docx]

Additional file 2

10 Things to Know about Adult Rheumatology Clinic

1. As an adult, you are in charge of your health care. Your doctor expects you to know your medications, how to request refills, and how to schedule appointments. If you do not know something, ask!
2. Call the clinic if you have questions, need refills, are having new symptoms, or want to leave a message for your doctor. Leave a message with a medical assistant and someone will call you back to discuss.
3. There is a rheumatologist “on-call” after business hours (nights and weekends). This doctor is NOT available for refills, routine questions, or issues that can wait until the next business day. This is a service for medical emergencies. If you do not know, ASK how to reach this “on-call” doctor.
4. Once you have transitioned from your pediatric rheumatologist to your adult rheumatologist, your adult rheumatologist will now prescribe your medications and order your tests. If you have questions, call your adult rheumatologist. If you have a medical emergency, you need to visit the adult emergency room.
5. During your clinic visit, cell phones should not be used. This is your time with your doctor. Phones should be off, or on silent, and put away during your visit.
6. To ensure that you are in charge of your own healthcare, your adult rheumatologist wants your visits to be “parent/caregiver free” for at least 50% of the visit. If you prefer a 100% “parent/caregiver free” visit, let your rheumatologist know. You are an adult and you have this right.
7. If you are more than 15 minutes late for your appointment, your visit will be canceled. In some cases, this may mean that you must wait many weeks before you can be seen. If you know that you are going to be late to your appointment or you need to reschedule your appointment, call your clinic (the sooner the better). Your adult rheumatologist knows that unexpected things happen, but the sooner that you let your clinic know that you will be late or that you will miss your visit, the easier it will be to re-schedule you sooner.
8. Your adult rheumatologist will want to examine you in a hospital gown at almost every visit. Please dress in appropriate undergarments to facilitate a gowned examination at every visit.
9. To avoid communication errors, you will be asked to choose a designated pharmacy and a designated contact phone number where you are comfortable with your adult rheumatologist leaving medical messages on you voice mail. If you wish to make changes to your phone number or pharmacy at any time, please let your clinic know. Many clinics also have online services that allow you to email your doctor confidentially. Ask about your options to ensure that you and your doctor can always reach each other.
10. When possible, labs, x-rays, and other tests will be performed through your adult rheumatology clinic and their facilities. This makes it easier for your adult rheumatologist to take care of you safely. Please be prepared to have tests performed when you visit your doctor.
